# Mortality Risk From COVID-19 Among Unvaccinated Subjects With Autoimmune Phenotypes of Interstitial Lung Disease

**DOI:** 10.7759/cureus.23808

**Published:** 2022-04-04

**Authors:** Rachel K Strykowski, Maria Poonawalla, Albina Tyker, Iazsmin Bauer Ventura, Cathryn Lee, Renea Jablonski, Rekha Vij, Jonathan Chung, Mary Strek, Ayodeji Adegunsoye

**Affiliations:** 1 Department of Medicine, Division of Pulmonary and Critical Care, University of Chicago Medicine, Chicago, USA; 2 Department of Medicine, University of Chicago Medicine, Chicago, USA; 3 Department of Medicine/Rheumatology, University of Chicago Medicine, Chicago, USA; 4 Department of Medicine/Pulmonary and Critical Care, University of Chicago Medicine, Chicago, USA; 5 Department of Radiology, University of Chicago Medicine, Chicago, USA

**Keywords:** immuno suppresion, interstital lung disease, auto immune, covid-19 pneumonia, covid 19

## Abstract

Background: The impact of the severe acute respiratory syndrome-associated coronavirus 2 (SARS-CoV-2) virus on patients with interstitial lung disease (ILD) remains poorly understood. As patients with ILD often have severe underlying lung parenchymal involvement, and immunosuppressive therapy is common in this population, they are presumed to be at high risk for severe coronavirus disease 2019 (COVID-19) pneumonitis. Our aim was to explore demographic and clinical differences between those with ILD who tested positive for the SARS-CoV-2 virus compared to those with ILD who did not.

Methods: In this retrospective cohort study, we identified adult, unvaccinated patients evaluated at the University of Chicago in 2020 who were enrolled in the ILD registry, and stratified by SARS-CoV-2 seropositive status. We then compared baseline clinical characteristics between SARS-CoV-2 seropositive and SARS-CoV-2 seronegative patients and assessed immunosuppressive therapy that the patient may have been on since ILD diagnosis. C-reactive protein and leukocyte subsets were evaluated at COVID diagnosis compared to the time of baseline ILD evaluation as were pulmonary function testing. Variable comparisons were determined by two-sided t-tests or chi-square tests as appropriate, and logistic regression models were fitted to assess the odds of death from COVID-19 using generalized linear models with maximum-likelihood estimation.

Results: Of the 309 individuals with ILD in our cohort, 6.8% (n=21) tested positive for SARS-CoV-2. Those who were SARS-CoV-2 positive were younger (57 years vs 66 years; P=0.002), had baseline higher total lung capacity (81% vs 73%, P=0.045), similar forced vital capacity (71% vs. 67%, P=0.37), and similar diffusion capacity of carbon monoxide (71% vs. 62%, P=0.10) at baseline. Among patients with ILD and COVID-19, 67% had received immunosuppressive therapies compared to 74% of those with ILD without COVID-19. Those with ILD and COVID-19 were also more likely to have had a diagnosis of autoimmune-related ILD (connective tissue disease-ILD or interstitial pneumonia with autoimmune features) (62% vs 38%, P=0.029). Overall, the mortality hazard was highest among unvaccinated subjects with autoimmune-related ILD who had COVID-19 (OR=9.6, 95% CI=1.7-54.0; P=0.01).

Discussion: SARS-CoV-2 is prevalent in ILD, and may put unvaccinated adults who are younger, with autoimmune ILD, and on immunosuppressive therapy at higher risk. This suggests a need for COVID-19 vaccinations and therapy (inpatient and outpatient) for this group of patients at high risk for COVID-19. Larger studies are needed to fully explore the relationship between ILD and immunosuppressive therapy in COVID-19.

## Introduction

Coronavirus disease 2019 (COVID-19) is an infectious disease caused by severe acute respiratory syndrome (SARS)-associated coronavirus 2 (SARS-CoV-2) and it remains an emergent threat to public health. The widespread systemic effect exerted by COVID-19 suggests an exuberant immune response. This is supported by recent investigations that demonstrate a causal relationship between immune dysregulation and the release of pro-inflammatory cytokines from SARS-CoV-2 infection [1-3]. Involvement of these pathogenic pathways has refocused the spotlight on immunomodulatory therapies for COVID-19, and the importance of vaccination to reduce mortality [4,5].

Interstitial lung diseases (ILDs) comprise a heterogeneous group of disorders [6,7]. A significant proportion of patients who have ILD will have an autoimmune-related form of the disease. These specific diagnoses include those associated with defined connective tissue disease (CTD-ILD) and those who show clinical or serologic features consistent with CTD without meeting established CTD diagnostic criteria, currently referred to as interstitial pneumonia with autoimmune features (IPAF) [7]. Many of these individuals receive immunosuppressive therapy. While these therapies are of great overall benefit, the higher propensity for systemic infections often puts patients at increased risk for acute ILD exacerbations and mortality [8].

Notably, infection with respiratory viruses such as SARS-CoV-2 increases mortality risk in ILD [9]. However, vaccination against respiratory viruses in patients with ILD effectively boosts the production of protective IgG antibodies [7,8]. Whether SARS-CoV-2 infection heightens the risk for mortality/poor outcomes in unvaccinated patients receiving immunosuppressive therapies for autoimmune-related ILD is unclear. Here, we performed a single-center retrospective study for the year 2020, prior to the widespread distribution of vaccines. We hypothesized that SARS-CoV-2 infection in unvaccinated patients receiving immunosuppressive therapy for autoimmune-related ILD would increase the risk of detrimental outcomes.

## Materials and methods

In this retrospective cohort study, we identified adult patients (age range of 19-86 years) unvaccinated for COVID-19, with a multidisciplinary diagnosis of ILD evaluated at the University of Chicago between January 1, 2020 and December 31, 2020, who were enrolled in the Natural History of ILD registry (IRB#14163A), and stratified by detection of the SARS-CoV-2 virus presence or absence on polymerase chain reaction (PCR). Demographic data and pulmonary function testing values were obtained at the time of ILD diagnosis. All patients were assessed for immunosuppressive therapies received since ILD diagnosis. Corticosteroid therapy was noted if ≥20 mg of prednisone or its equivalent was given for at least three months. Vital status was determined from a chart review and the social security death index.

Statistical analysis

We conducted hypothesis testing using SARS-CoV-2 virus presence or absence on PCR as a binary variable to determine prevalence across predefined features of the ILD cohort. Variable comparisons were determined by two-sided t-tests, Mann-Whitney U tests, or chi-square tests as appropriate. To examine the association of SARS-CoV-2 and all-cause mortality in patients with ILD, the presence of autoimmune-related ILD was treated as a binary variable, and duration of follow-up was treated as a continuous variable. We calculated survival time as time from baseline ILD evaluation to death, lung transplantation, loss to follow-up, or end of the study period. Survival time was censored on April 30, 2021 or if lost to follow-up. Logistic regression models were fitted for the assessment of the mortality outcome using generalized linear models with maximum-likelihood estimation (2019.R.16; StataCorp, College Station, TX).

## Results

Of the 309 individuals with ILD in our cohort, 6.8% (n=21) tested positive for SARS-CoV-2. Those who were SARS-CoV-2 positive were younger (57 years vs 66 years; P=0.002), had baseline higher total lung capacity (81% vs 73%, P=0.045), similar forced vital capacity (71% vs 67%, P=0.37), and similar diffusion capacity of carbon monoxide (71% vs 62%, P=0.10) at baseline, similar prevalence of honeycomb fibrosis (43% vs 30%; P=0.22), similar baseline leukocyte count (9.4 vs 8.4; P=0.27), and marginally lower C-reactive protein (CRP) (4.9 vs 9.3; P=0.07) (Table 1). Except for body mass index, n=223, and the subset in which circulating leukocyte count was evaluated (n=89), missing data were less than 5% for all clinical features assessed.

**Table 1:** Demographic characteristics of interstitial lung disease cohort. TLC=total lung capacity; FVC=forced vital capacity; SARS-CoV-2=severe acute respiratory syndrome coronavirus 2; CAD=coronary artery disease; DM=diabetes mellitus; FEV_1_=forced expiratory volume in 1 second; CRP=c-reactive protein; DLCO=diffusion capacity of carbon monoxide; CTD=connective tissue disease; ILD=interstitial lung disease; IPAF=interstitial pneumonia with autoimmune features; WBC=white blood cell.

Characteristics of ILD cohort (n=309)	COVID-19 positive (n=21)	COVID-19 negative (n=288)	P-value
Age (years), mean (±SD)	57.3 (16)	66.0 (12)	0.002
Male gender, n (%)	7 (33)	111 (39)	0.64
Race			
Caucasian, n (%)	11 (52)	131 (58)	0.59
BMI, mean (±SD)	30.2 (5)	31.0 (7)	0.61
Ever smoker, n (%)	8 (38)	127 (44)	0.59
Smoking, pack-years, mean (±SD)	5 (9)	10 (18)	0.21
Gastroesophageal reflux, n (%)	14 (67)	173 (60)	0.55
Emphysema, n (%)	4 (19)	81 (28)	0.36
CAD, n (%)	3 (14)	48 (17)	0.78
DM, n (%)	5 (24)	63 (22)	0.89
Hypothyroidism, n (%)	5 (24)	46 (16)	0.35
TLC (% predicted) (±SD)	80.1 (16)	73.0 (17)	0.045
FVC (% predicted) (±SD)	71.2 (17)	67.4 (18)	0.37
FEV_1_ (% predicted) (±SD)	71.2 (19)	76.7 (16)	0.21
DLCO (% predicted) (±SD)	70.9 (18)	61.6 (23)	0.1
Radiologic honeycombing, n (%)	9 (43)	86 (30)	0.22
CTD-ILD/IPAF, n (%)	13 (62)	109 (38)	0.029
Corticosteroid therapy, n (%)	13 (62)	150 (52)	0.38
ANA seropositivity, n (%)	8 (42)	134 (49)	0.56
CRP titer, mean (±SD)	4.9 (2.2)	9.2 (9.6)	0.07
WBC, mean (±SD)	9.4 (9)	8.4 (3)	0.27
Lymphocyte %, mean (±SD)	28 (17)	21 (10)	0.029
Died, n (%)	3 (14)	11 (4)	0.026

**Figure 1:**
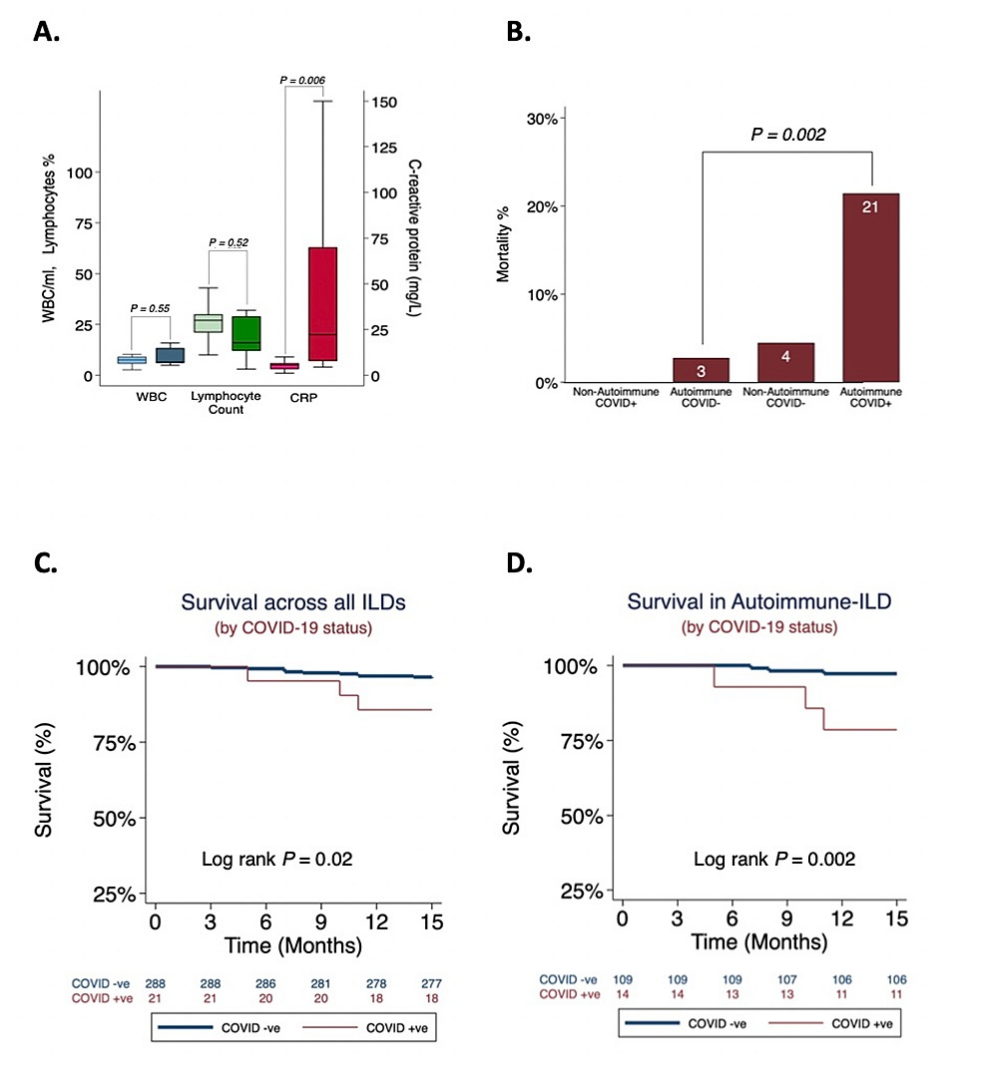
Cell counts and mortality in COVID-19 (A) Change in WBC count, peripheral lymphocyte fraction (%), and CRP titers before and after diagnosis of COVID-19 in subjects with interstitial lung disease. (B) Mortality is highest among COVID-positive subjects with autoimmune-related ILD. (C) Survival pattern by COVID-19 status across all ILDs. (D) Survival pattern by COVID-19 status across autoimmune-related ILDs. WBC=white blood cell; CRP=C-reactive protein; ILD=interstitial lung disease; COVID-19=coronavirus disease 2019.

Among patients with ILD and COVID-19, 67% had ever received immunosuppressive therapies compared to 74% of those with ILD without COVID-19. Those with ILD and COVID-19 were also more likely to have had a diagnosis of autoimmune-related ILD (CTD-ILD or IPAF) (62% vs 38%, P=0.029), higher baseline lymphocyte counts (28% vs 21%, P=0.025), and were more frequently hypoxemic (SpO_2_ ≤ 92%; 4% vs 19%; P=0.012) at ILD diagnosis than those without COVID-19. The majority of patients (62%) with COVID-19 had received lymphocyte-depleting immunosuppressive therapy (prednisone, azathioprine, mycophenolate, rituximab) prior to infection. At the time of SARS-CoV-2 detection, CRP titers were much higher (52 mg/L vs 5mg/L; P=0.006) than at ILD diagnosis; however, the lymphocyte fraction was not different (24% vs 28%; P=0.52) (Figure 1A). Not surprisingly, unvaccinated ILD subjects with COVID-19 had higher odds of death than those without COVID-19 (OR=4.2, 95%CI=1.1-16.4; P=0.039). Interestingly, the mortality hazard was highest among unvaccinated subjects with autoimmune-related ILD who had COVID-19 (OR=9.6, 95%CI=1.7-54.0; P=0.01) (Figure 1B-1D).

## Discussion

Our study examines the association of SARS-CoV-2 positivity in unvaccinated patients with underlying ILD. Overall, 6.8% of our unvaccinated ILD population tested positive for SARS-CoV-2 in 2020, which is comparable to state-wide prevalence rates in Illinois in 2020 of about 7.5% (963,389 cases with a population of around 12.8 million) [10]. These findings differ from a nationwide study in Korea done by Lee H et al. (2021) [11], who found an increased risk of COVID-19 in those with ILD. Specifically, that study used a nested case-control model and found that the proportion of patients with ILD was significantly higher in the COVID-19 cohort (total n=8070) compared to the matched cohort (total n=121,050) (0.8% vs 0.4%, P<0.001). The differences in these results could be attributed to the present study’s small sample size.

However, this study did have some interesting and novel findings. Specifically, it found that unvaccinated patients with ILD who tested positive for COVID-19 were younger and were more likely to have an autoimmune-related ILD (CTD-ILD or IPAF). While slightly controversial, some studies have shown that patients with autoimmune disease have an increased risk of COVID-19 [11,12]. However, this is the first study, to our knowledge, that shows that patients with autoimmune-related ILD are at even higher risk for COVID-19 compared to those with other ILDs. Further, this study found that subjects with COVID-19 and ILD had higher odds of death compared to those without, with the highest prevalence of mortality among those with autoimmune ILD. Interestingly, these findings reverse the favorable prognostic patterns commonly seen with autoimmune ILDs compared to other types of ILD [12-14]. One such single-center study (ILD, n=305) by Lim et al. (2019) [14] found that compared to other ILD groups those with IPAF had better survival and fewer episodes of exacerbation when compared to the IPF group.

Over half (62%) of our patients who tested positive for SARS-CoV-2 had received lymphocyte-depleting immunosuppressive therapy. This is not entirely surprising given that previous studies have shown that in patients, with iatrogenic B-cell depletion, particularly with agents targeting CD-20, there is an increased risk of severe COVID-19 and death across a wide range of different disease states, and these patients also have a more challenging course with seroconversion after primary infection [15,16]. However, interestingly in our present study, immunosuppressive therapy was numerically higher in the ILD without COVID group compared to the COVID with ILD (74% vs 67%) though not statistically significant. Overall our data suggest that the risk for these patients may not be only attributable to being on immunosuppressive therapy, but that the underlying chronic autoimmune lung disease may accentuate their risk.

Additionally, those with COVID-19 and ILD were more frequently hypoxemic. Beyond case reports, to our knowledge, it is not clear if patients have more prolonged hypoxia when they have COVID-19 and ILD [17]. However, there is some evidence that asymptomatic hypoxia in COVID-19 is associated with poor outcomes [18]. Thus, future studies may look at the subjective complaint of hypoxia in our patients with ILD and COVID-19 along with associated outcomes. Although the prevalence of honeycomb fibrosis was numerically higher, this was not statistically significant. This suggests that patients with ILD who are at the highest risk of COVID-19 may have a more aggressive autoimmune phenotype morphologically characterized by honeycombing fibrosis, and more profound hypoxemia.

There are some limitations to this study, notably the small sample size (COVID-19+ with n=21) and the timing of the study which pre-dates COVID vaccinations. This makes the applicability of this study slightly limited to patients with COVID-19 who have ILD and are not vaccinated. Future studies could compare larger sample sizes and look at the effect of vaccination on outcomes stratified by immunosuppressive medication. Further, we evaluated the use of immunosuppressive medications since diagnosis of ILD and did not note whether or not the patient was on the medication at the time of COVID-19 diagnosis. Future studies could explore medication use concurrently with COVID-19 with more granularity [19,20].

## Conclusions

This study lends credence to the idea that immunosuppressed patients with ILD, particularly those on lymphocyte-depleting immunosuppressive therapy, are a population in which vaccinations and non-pharmaceutical interventions should be prioritized for the prevention of COVID-19. Further, given the elevated risk, this group is increasingly being considered for therapeutic outpatient and inpatient pharmacologic treatment of COVID-19, and this study further supports these interventions.
